# Forensic postmortem examination as sentinel evidence of rifampicin-resistant tuberculosis outside routine clinical reporting

**DOI:** 10.1016/j.ijregi.2026.100924

**Published:** 2026-05-23

**Authors:** Nathan M. Kayonde, Bwalya Mulenga, Cordilia M. Himwaze, Chibamba N. Mumba, Vutisa Dokowe, Chitala Chingoli, Viktor Telendiy, Luce Mazyopa, Thato M. Patlakwe, Linzy Elton, John Tembo, Timothy D. McHugh, Francine Ntoumi, Luchenga A. Mucheleng'anga

**Affiliations:** 1 Office of the State Forensic Pathologist, Lusaka Provincial Forensic Pathology Unit, University Teaching Hospitals, Lusaka, Zambia; 2 Department of Pathology and Microbiology, School of Medicine, The University of Zambia, Lusaka, Zambia; 3 Faculty of Forensic Pathology, Zambia College of Medicine and Surgery, Lusaka, Zambia; 4 University Teaching Hospitals‒Adult Hospital, Lusaka, Zambia; 5 Centre for Clinical Microbiology, University College London, London, UK; 6 PANDORA-ID-NET Pathogenesis Group, and UNZA-UCLMS Research and Training Program, University Teaching Hospital, Lusaka, Zambia; 7 Centre for Innovation in Genomics and Microbiome Studies, University of West London, London, UK; 8 Foundation Congolaise pour la Recherche Médicale (FCRM), Brazzaville, Republic of Congo; 9 Institute of Tropical Medicine, University of Tübingen, Tübingen, Germany

**Keywords:** Rifampicin-resistant tuberculosis, Postmortem examination, Community deaths, GeneXpert MTB/RIF Ultra, Sentinel surveillance

## Abstract

**Objectives:**

Drug-resistant tuberculosis (TB) is underascertained when deaths occur outside health facilities. We evaluated whether forensic postmortem molecular testing identifies rifampicin-resistant TB among community deaths not represented in routine clinical reporting.

**Methods:**

We conducted a prospective observational study of community deaths receiving medico-legal postmortem examination in Lusaka, Zambia (September 2022-April 2024). Cases with macroscopic features suggestive of TB underwent histologic evaluation, and histology-compatible cases were tested using GeneXpert MTB/RIF Ultra.

**Results:**

Among 2988 postmortem examinations, 73 decedents had histologic features consistent with TB. *Mycobacterium tuberculosis* deoxyribonucleic acid was detected in 56/73 cases, with rifampicin resistance identified in eight/56.

**Conclusion:**

Forensic postmortem molecular testing identified rifampicin-resistant TB among community deaths not represented in routine clinical drug-resistance reporting. Although not informative of prevalence, these findings indicate that postmortem examination can provide sentinel evidence of drug-resistant TB beyond conventional diagnostic pathways.

## Highlights

• Rifampicin-resistant tuberculosis was identified among community deaths at postmortem examination.
• GeneXpert MTB/RIF Ultra detected resistance in histology-confirmed postmortem tissue.
• Postmortem testing provides sentinel evidence of drug-resistant tuberculosis beyond clinical reporting.

## Introduction

Drug-resistant tuberculosis (TB) remains a major challenge in high-burden settings, where delayed diagnosis and incomplete access to microbiological testing undermine control efforts [1]. Routine surveillance depends on patients reaching health services; deaths occurring outside clinical care, including those referred for medico-legal postmortem examination, are therefore commonly absent from diagnostic and drug-resistance reporting systems.

In sub-Saharan Africa, many deaths occur outside health facilities, and autopsy studies have shown substantial undiagnosed TB in hospital and community deaths [[2], [3], [4], [5], [6]]. This creates a surveillance blind spot, especially in high human immunodeficiency virus (HIV)/TB burden settings. Postmortem examination cannot estimate population prevalence, but it can provide sentinel evidence of disease occurring beyond clinical surveillance.

Molecular testing of postmortem tissue can detect *Mycobacterium tuberculosis* deoxyribonucleic acid (DNA) when histology or staining result is negative [7]. We therefore applied GeneXpert MTB/RIF Ultra to postmortem-confirmed TB among community deaths in Lusaka, Zambia, to determine whether forensic postmortem molecular testing could identify rifampicin-resistant TB not represented in routine clinical drug-resistance reporting.

## Methods

We conducted a study of deaths that underwent postmortem examination at the Lusaka Forensic Pathology Unit, Zambia, from September 2022 to April 2024. Community deaths were referred under the Inquests Act (Cap. 36), including deaths without a Certificate of Cause of Death under the Births and Deaths Registration Act (Cap. 51). This population included deaths with unknown cause.

All examinations followed a postmortem protocol under the Office of Forensic Pathologist Practice Manual (2018), including examination of lungs, liver, spleen, kidneys, and lymphatic tissues. Macroscopic assessment for TB was performed by specialist pathologists. Macroscopic features suggestive of TB, including lymph node involvement, triggered targeted sampling. Cases were included when histologic evaluation showed necrotizing granulomatous inflammation. Decomposition or infeasible sampling were the exclusion criteria.

The histologic examination used hematoxylin-eosin and Ziehl-Neelsen staining. Tissue from all histologically confirmed cases underwent GeneXpert MTB/RIF Ultra testing for *Mycobacterium tuberculosis* DNA and rifampicin resistance. Necrotizing granulomatous inflammation was interpreted, particularly in individuals who were immunocompromised. Cases showing suppurative necrosis without granulomatous inflammation were excluded from molecular testing, which may have underestimated TB in decedents. GeneXpert was considered confirmation when DNA was amplifiable.

Age, sex, and HIV status were abstracted from postmortem records. HIV status was obtained from clinical documentation or rapid testing when unavailable. Examinations were performed by two forensic pathologists, one anatomical pathologist, and two medical officers.

Data were analyzed using counts, proportions, medians, and ranges. Binomial confidence intervals were calculated for rifampicin resistance among GeneXpert-positive cases. Postmortem examinations were authorized under the Inquests Act. Molecular testing and analysis of data were approved by the University of Zambia Biomedical Research Ethics Committee (4087-2023) and the National Health Research Authority.

## Results

**Figure 1:**
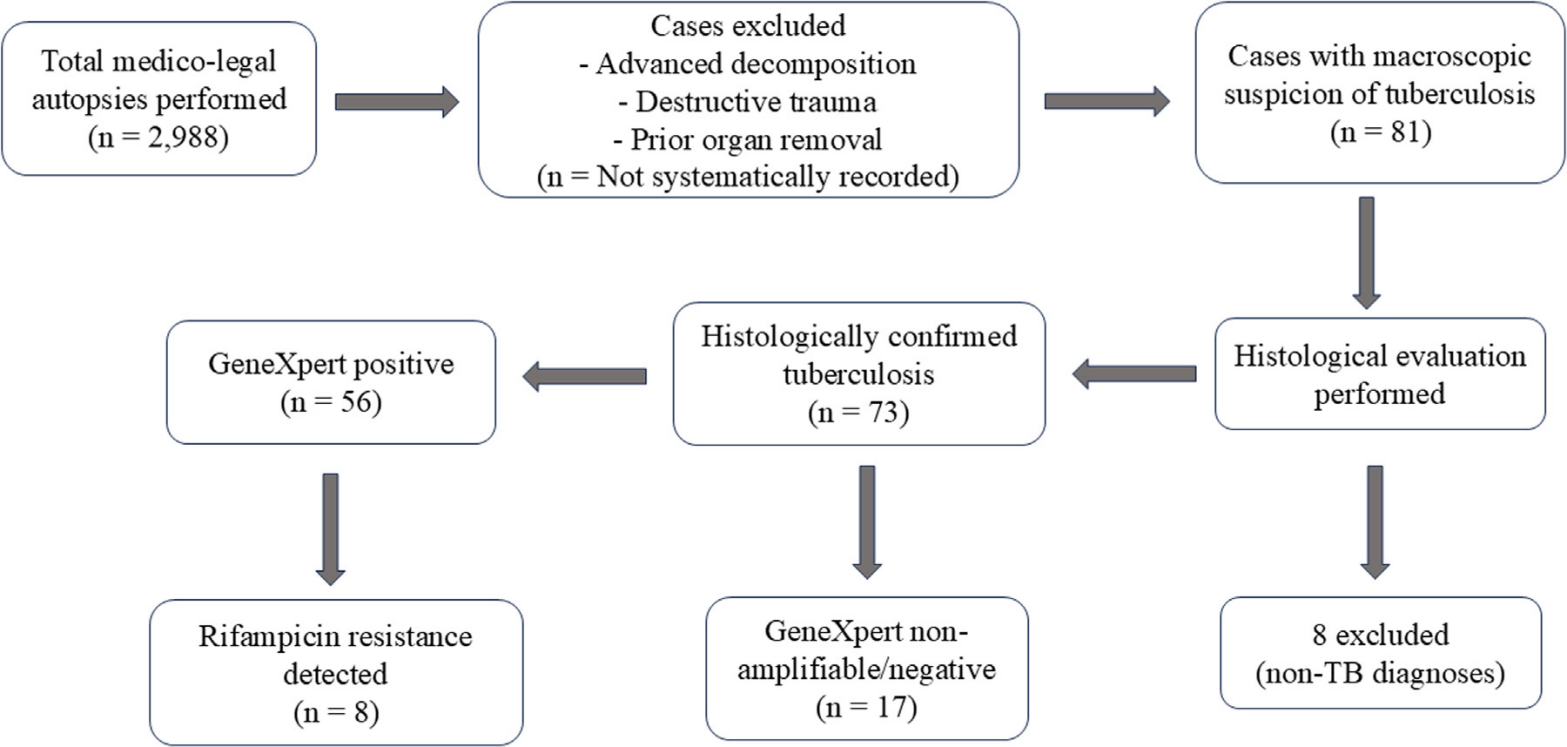
Study flow diagram showing screening, histologic confirmation, exclusions, GeneXpert MTB/RIF Ultra testing outcomes, and rifampicin-resistance detection among community deaths receiving medico-legal postmortem examination in Lusaka, Zambia.

During the study period, 2988 community deaths underwent postmortem examination. Of 81 cases with macroscopic features of TB, 73 were confirmed histologically and included, whereas eight were excluded. All 73 included cases underwent GeneXpert testing; 56 yielded amplifiable *Mycobacterium tuberculosis* DNA, whereas 17 did not and were not assessable for rifampicin resistance (Figure 1).

Among the 2988 examinations, 2376 (80%) were in male and 612 (20%) in female patients. Among the 81 macroscopically suspected cases, 71 (88%) were in male and ten (12%) in female patients. Among the 73 histologically confirmed TB cases, 65 (89%) were in male and eight (11%) in female patients. Eight cases showed macroscopic appearances suggestive of TB, but necrotizing granulomatous inflammation on histologic evaluation was absent, and these were excluded. Histologic diagnoses in excluded cases were bacterial pneumonia (n = 5), lung abscess (n = 2), and lung infarction (n = 1) (Table 1).

**Table 1:** Postmortem detection and molecular findings of TB among community deaths (N = 2988).

Characteristic	n (%)
**Overall study population**	
Total community deaths examined	2988
Male sex	2376 (80%)
Female sex	612 (20%)
**Macroscopic screening (n = 2988)**	
Cases with suspicion of TB	81 (2.7%)
**Histologic evaluation (n = 81)**	
Histology-confirmed TB	73 (90.1%)
Excluded (non-TB diagnoses)	8 (9.9%)
Bacterial pneumonia	5
Lung abscess	2
Lung infarction	1
**Histology-confirmed TB (n = 73)**	
Male sex	65 (89%)
Female sex	8 (11%)
TB-related causes of death	49 (67.1%)
Non-TB causes of death	24 (32.9%)
Ziehl-Neelsen positive	47 (64.4%)
Ziehl-Neelsen negative	26 (35.6%)
GeneXpert MTB/RIF Ultra positive	56 (76.7%)
GeneXpert non-amplifiable	17 (23.3%)
**GeneXpert-positive cases (n = 56)**	
Median age, y (range)	35 (19-60)
Male sex	50 (89%)
HIV positive	34 (61%)
Ziehl-Neelsen positive	47 (83.9%)
Ziehl-Neelsen negative	9 (16.1%)
**Rifampicin resistance (n = 56)**	
Rifampicin-resistant	8 (14.3%)
Rifampicin-susceptible	48 (85.7%)
Ziehl-Neelsen positive among resistant cases	7/8 (87.5%)
Ziehl-Neelsen negative among resistant cases	1/8 (12.5%)

TB was defined by necrotizing granulomatous inflammation on histologic evaluation. Cases with macroscopic suspicion but lacking histologic confirmation were excluded. GeneXpert MTB/RIF Ultra was performed on all histology-confirmed cases; non-amplifiable results were attributed to postmortem DNA degradation. Rifampicin resistance was assessed only in cases with amplifiable DNA. Percentages are calculated within specified denominators. Table 1 summarizes postmortem TB detection and molecular findings among 2988 community deaths. Of these, 81 cases (2.7%) showed macroscopic features suggestive of TB and underwent histologic evaluation, of which 73 (90.1%) were confirmed and eight (9.9%) excluded because of alternative diagnoses. Ziehl-Neelsen staining was positive in 47/73 cases (64.4%). GeneXpert MTB/RIF Ultra detected *Mycobacterium tuberculosis* DNA in 56 cases (76.7%), whereas 17 (23.3%) were non-amplifiable. Among GeneXpert-positive decedents, the median age was 35 years (range 19-60); 50 (89%) were male, and 34 (61%) were HIV positive. Rifampicin resistance was identified in eight/56 cases (14.3%).

Abbreviations: DNA, deoxyribonucleic acid; HIV, human immunodeficiency virus; TB, tuberculosis.

Among the 73 decedents with identified TB, 49 had TB-related causes of death, comprising pulmonary TB (n = 34), disseminated TB (n = 13), pleural empyema in a decedent with pulmonary TB (n = 1), and peritonitis in a decedent with disseminated TB (n = 1). In 24 cases, TB was identified histologically, but the primary cause of death was non-TB, including community-acquired pneumonia (n = 6), alcohol intoxication (n = 4), toxic ingestion (n = 2), acquired immunodeficiency syndrome (n = 2), trauma (n = 2), and other categories.

Ziehl-Neelsen staining was positive in 47/73 cases and negative in 26/73. GeneXpert MTB/RIF Ultra testing was performed on tissue from 73 histology-confirmed cases; 56 yielded amplifiable *Mycobacterium tuberculosis* DNA, and 17 did not. The 56 amplifiable cases were histology-compatible and GeneXpert-confirmed TB cases.

Among these 56 dead patients showing GeneXpert-positive, the median age was 35 years (range 19-60); 50/56 (89%) were male, and HIV infection was in 34/56 (61%). Of the 56 GeneXpert-positive cases, 47 were Ziehl-Neelsen positive and nine negative, indicating that nine/56 (16%) cases would not have been detected by Ziehl-Neelsen staining. All 17 cases were Ziehl-Neelsen negative.

Rifampicin resistance was identified in eight/56 GeneXpert-positive cases (14.3%, 95% confidence interval 6.4-26.9). Seven rifampicin-resistant cases were Ziehl-Neelsen positive and one Ziehl-Neelsen negative. Because resistance could only be assessed in cases with amplifiable DNA, the proportion of drug-resistant TB among the 73 histology-compatible cases could not be determined.

Rifampicin-resistant cases occurred across age categories, with no clustering by sex or HIV status. No indeterminate rifampicin-resistance results occurred.

## Discussion

The detection of rifampicin-resistant TB in this study indicates disease occurring outside diagnostic pathways and provides sentinel evidence of drug-resistant TB beyond clinical reporting. In Zambia, drug-resistant TB remains a public-health challenge [1,[8], [9], [10]]. Resistance rates are 2.8% in new cases and up to 18% in previously treated TB [9], alongside an HIV co-infection burden of 34% [10]. Autopsy data from Lusaka showing incidental TB in 5% support undiagnosed disease at death [5]. Detection of rifampicin-resistant pulmonary TB suggests disease may have remained undiagnosed before death.

Case identification relied on macroscopic suspicion followed by histologic confirmation, capturing disease. These findings represent a selectively ascertained subset of community TB deaths. Most histologically identified cases had TB-related certified causes of death, but one-third had non-TB primary causes, showing that postmortem examination can identify fatal and unreported co-existing TB.

Rifampicin resistance was identified in eight of 56 GeneXpert-positive cases. This proportion applies only to GeneXpert-positive cases and should not be interpreted as prevalence among TB deaths. Because resistance could only be assessed in cases with molecular material, the true proportion of rifampicin resistance among histology-compatible cases may be underestimated.

A total of 16% of GeneXpert-confirmed cases were Ziehl-Neelsen negative, indicating that reliance on histologic staining alone would have failed to detect infections. This supports the role of molecular testing in postmortem investigation [7]. The 17 histology-compatible cases without amplifiable DNA reflect limitations of postmortem diagnostics.

Community deaths outside routine pathways may be missed by clinical drug-resistance reporting systems [6]. Although examination cannot address this gap at scale, molecular testing may provide sentinel evidence of disease.

This study has limitations. Case identification relied on macroscopic suspicion and histologic confirmation and may have missed non-granulomatous TB. Histologic classification without molecular confirmation may have included non-tuberculous granulomatous disease. Eight cases were excluded after histologic evaluation. DNA degradation limited resistance assessment in some decedents [7]. TB history was not consistently available, preventing distinction between primary and acquired resistance.

## Conclusion

Forensic postmortem molecular testing identified rifampicin-resistant TB among community deaths not represented in routine clinical reporting. The findings do not estimate rifampicin-resistant TB prevalence, but indicate the value of postmortem examination as a sentinel indicator of drug-resistant TB outside conventional diagnostic pathways.

## CRediT authorship contribution statement

**Nathan M. Kayonde:** Conceptualization, Data curation, Formal analysis, Investigation, Methodology, Project administration, Resources, Software, Supervision, Validation, Visualization, Writing – original draft, Writing – review & editing. **Bwalya Mulenga:** Formal analysis, Investigation, Methodology, Validation, Writing – original draft, Writing – review & editing. **Cordilia M. Himwaze:** Data curation, Investigation, Supervision, Writing – original draft, Writing – review & editing. **Chibamba N. Mumba:** Data curation, Formal analysis, Investigation, Methodology, Supervision, Writing – original draft, Writing – review & editing. **Vutisa Dokowe:** Writing – original draft, Writing – review & editing. **Chitala Chingoli:** Writing – original draft, Writing – review & editing. **Viktor Telendiy:** Writing – original draft, Writing – review & editing. **Luce Mazyopa:** Writing – original draft, Writing – review & editing. **Thato M. Patlakwe:** Writing – original draft, Writing – review & editing. **Linzy Elton:** Writing – original draft, Writing – review & editing. **John Tembo:** Writing – original draft, Writing – review & editing. **Timothy D. McHugh:** Writing – original draft, Writing – review & editing. **Francine Ntoumi:** Writing – original draft, Writing – review & editing. **Luchenga A. Mucheleng'anga:** Conceptualization, Data curation, Formal analysis, Investigation, Methodology, Supervision, Validation, Writing – original draft, Writing – review & editing.

## Declaration of competing interest

The authors have no competing interests to declare.
